# 

**Published:** 1865-11

**Authors:** 


### SUBJECTS FOR DISCUSSION.

### SOCIETY OF DENTAL SURGEONS OF THE CITY OF NEW YORK.

[1865.]

Sept. 27,—Congenital Cleft Palate.
Oct. 11,—Dental Education.
" 25,—Origin of the Teeth.
Nov. 8,—Development of the Teeth.
" 23,—Pathology of the Teeth.
Dec. 6,—Hypertrophy of the Cement.
" 20,—Cause and Treatment of Caries.

[1866.]

Jan. 3,—Sympathetic Diseases of the Teeth.
" 17,—Impressions, Models and Dies.
" 31,—Vulcanite.
Feb. 14,—Pivot Teeth.
" 28,—Dental Instruments.
Mar. 14,—Materials for Filling Teeth.
" 28,—Dental Medicines.
April 11,—Keeping Cavities Dry.
" 25,—Filling Special Cavities, Buccal and Root.
May 9,—Cause and Treatment of Irregularities.
" 23,—Disease of the Antrum.
June 6,—The Mallet.
" 20,—Glasses in Dental Operations.
July 4,—Cause and Treatment of Epulis.
" 18,—Dental Associations.

JNO. M. CROWELL,

FRANK ABBOTT,

WM. H. ATKINSON, } Committee.

### BROOKLYN DENTAL ASSOCIATION.

[1865.]

Oct. 4,—Congenital Cleft Palate.

" 18,—Anatomy and Physiology of the Mouth.

Nov. 1,—Pathological Conditions of the Mouth.

" 15,—Anatomy and Physiology of the Teeth.

Nov. 29,—Absorption of the Alveoli.
Dec. 13,—Preservation of Exposed Pulps.
" 27,—Horizontal Grooving of the Teeth.

[1866.]

Jan. 10,—Alveolar Abscess.
" 24,—Continuous Gum.
Feb. 7,—Restoring Normal Expression of the Face.
" 21,—Clasps for attaching Plates.
Mar. 7,—Mineral Teeth.
" 21,—The Dentist's Duty to his Patient.
Apr. 4,—Dentifrices.
" 18,—Anæsthetics in Dentistry.
May 2,—Preparation of Cavities.
" 16,—Filling Proximal, Grinding and Compound Cavities.
" 30,—Preservation of Deciduous Teeth and Six-year Molars.
June 13,—Sensitive Dentine and Treatment.
" 27,—Necrosis of Alveolar Process and Treatment.
July 11,—Devitalizing and Removing Dental Pulps.

B. W. FRANKLIN, C. E. FRANCIS, J. S. LATIMER, } Committee.

### PECUNIARY.

We shall, in the December number of the Register, send out bills to all of our subscribers who are in arrears. We hope this notice will be the means of relieving us of much, or all of that work, by the very easy and simple matter of remitting us $3, before that time. Our printer and paper dealer, still insist upon having their bills paid ; we have frequently paid them when the Register had nothing to pay them with. A word to the wise is sufficient.

### SUBJECTS OF DISCUSSION BY THE CINCINNATI DENTAL ASSOCIATION.

1865.

Nov. 16— Absorption of the Alveoli.
" 30— Dental Education.
Dec. 14 — Alveolar Abscess.
" 28 — Treatment of Exposed Pulps.

1866.

Jan. 11 — Disease of the Antrum.
" 25 — Treatment of Sensative Dentine.
Feb. 8 — Dental Materia Medica.
" 22 — Treatment of Irregularities.
Mar. 8 — Sympathetic Diseases of the Teeth.
" 22 — Dental Associations.
April 5 — Pivot Teeth.
" 19 — Bleaching Teeth.
May 3 — Duty of the Dentist to his patient.
" 17 — Devitalization and removal of Dental Pulps.
" 31 — Mechanical Dentistry.

A. BERRY, }

J. TAFT, } Committee.

### Editorial.

### PERSONAL.

It is with the greatest satisfaction and gladness that we are able to state that Prof. WATT has so far recovered from his recent severe illness as to give some attention to business.

His power of locomotion is still rather feeble, yet constantly improving, though not rapidly. His condition is such however, that his ability to use the pen is better, he thinks, than for a long time previous to his illness, and in evidence of this, we have only to state that the prospect now is, that the profession will hear from him, through the pages of the journals far more frequently, than for some time past.

In the December number of the Register, he begins a series of articles, on "Dental Materia Medica," which is designed to embrace a consideration of all the medicinal agents pertaining especially to dental practice.

A treatise upon this subject is perhaps more needed by the profession, than upon any other single topic; as comparatively little attention has been given to it, and we know of no one in the profession, better able to treat the subject properly than Dr. WATT.

### The Dental Register

### OF THE WEST.

*Issued Monthly, at $3 a Year in Advance.*

DEVOTED TO THE INTERESTS OF THE DENTAL PROFESSION.

EDITED BY J. TAFT

The volume commences in January and closes in December.

The Register being issued monthly is always fresh, therefore indispensable to the practitioner who desires to keep posted up with the new inventions and improvements which are daily developed. Neither expense nor effort will be spared to give value and variety to its contents. Articles will be illustrated with well executed engravings whenever they will add to the interest or assist in explanation.

Its extensive circulation and frequency of issue makes it one of the BEST DENTAL ADVERTISING MEDIUMS in the United States.

RATES OF ADVERTISING.

One page, 1 year, $50. Half page, 1 year, $30. Quarter page, or card, 1 year $20.

All advertising bills payable in advance, or at furthest after the issue of the first number containing the advertisement. All transient advertisements to be paid for in advance.

☞ CONTRIBUTIONS SOLICITED.

Address, J. TAFT, or "DENTAL REGISTER," Cincinnati, Ohio.

Business Communications to

J. TAFT, Publisher,
No. 172 Race Street.

### ROBERTS' OS-ARTIFICIAL.

A substitute for AMALGAM in filling badly decayed teeth; and used for reacting Pivot Teeth in badly decayed roots, also for filling over SENSITIVE DENTINE to destroy sensibility, and as a non-conductor of heat, and for many other dental purposes.

For sale by all dealers in Dental Materials, and by the undersigned.

One-fourth ounce packages with directions sent by mail free of postage on receipt of $1.

C. H. ROBERTS, M. D.

